# Additive-Free Rice Starch-Assisted Synthesis of Spherical Nanostructured Hematite for Degradation of Dye Contaminant

**DOI:** 10.3390/nano8090702

**Published:** 2018-09-08

**Authors:** Juan Matmin, Irwan Affendi, Salizatul Ilyana Ibrahim, Salasiah Endud

**Affiliations:** 1 Centre of Foundation Studies UiTM, Universiti Teknologi MARA (UiTM), Cawangan Selangor, Kampus Dengkil, 43800 Dengkil, Selangor, Malaysia; affendi7848@puncakalam.uitm.edu.my (I.A.); saliza2910@puncakalam.uitm.edu.my (S.I.I.); 2 Chemistry Department, Faculty of Science, Universiti Teknologi Malaysia (UTM), 81310 Skudai, Johor, Malaysia; salasiah@kimia.fs.utm.my

**Keywords:** iron oxide, rice starch, templating method, hematite, additive-free synthesis, spherical structures, nanoparticles, nanostructured materials

## Abstract

Nanostructured hematite materials for advanced applications are conventionally prepared with the presence of additives, tainting its purity with remnants of copolymer surfactants, active chelating molecules, stabilizing agents, or co-precipitating salts. Thus, preparing nanostructured hematite via additive-free and green synthesis methods remains a huge hurdle. This study presents an environmentally friendly and facile synthesis of spherical nanostructured hematite (Sp-HNP) using rice starch-assisted synthesis. The physicochemical properties of the Sp-HNP were investigated by Fourier-transform infrared spectroscopy (FTIR), X-ray diffraction (XRD), thermogravimetric analysis (TGA), field emission scanning electron microscopy (FESEM), energy dispersive X-ray spectroscopy (EDX), transmission electron microscopy (TEM), UV-Vis diffuse reflectance spectroscopy (DR UV-Vis), and nitrogen adsorption–desorption analysis. The Sp-HNP showed a well-crystallized structure of pure rhombohedral phase, having a spherical-shaped morphology from 24 to 48 nm, and a surface area of 20.04 m^2^/g. Moreover, the Sp-HNP exhibited enhanced photocatalytic degradation of methylene blue dye, owing to the large surface-to-volume ratio. The current work has provided a sustainable synthesis route to produce spherical nanostructured hematite without the use of any hazardous agents or toxic additives, in agreement with the principles of green chemistry for the degradation of dye contaminant.

## 1. Introduction

Iron oxide nanoparticles (IONPs) in the form of magnetite, maghemite, lepidochrocite, goethite, akageneite, and hematite have emerged in advanced applications of medical processes, biotechnology, sensors, data storage, pigments, fine ceramics, photo/electrochemical cells, wastewater treatment, and catalysts [1,2,3,4]. Amongst all IONPs, hematite nanoparticles (HNPs) show the most prominent catalytic activity for organic transformation or degradation reactions, depending on the shape synthesized. The potential of a HNP can be further enhanced as a high-performance catalyst, which delivers good conversion activity, specific selectivity, and reliable stability simply by changing the exposed crystal facet via shape tailoring [5]. In recent years, a variety of synthetic methods have been investigated to produce HNPs using different approaches of physical synthesis (laser ablation arc discharge, combustion, ultrasound irradiation, electrodeposition, and pyrolysis) [6], biological synthesis (plant extracts, bacterium, yeast, fungus, and algae) [7] and chemical methods (sol–gel synthesis, the reverse micelle method, and hydrothermal methods) [8]. However, these methods have mainly yielded disordered or irregular-shaped nano/macro-sized HNP morphologies that create severe agglomeration, such as bitruncated polygons [8,9] and pseudo-peanut shapes [10]. Therefore, significant efforts have been devoted to controlling the morphology of HNPs by employing different synthesis and processing routes. Today, HNPs are prepared in different regular shapes and defined morphologies, such as nanocuboids, nanowhiskers, nanorods, ovals, cubes, rhombohedral shapes, and spherical morphologies [11,12]. Nevertheless, most of the preparation methods are less favored due to the complexity of the optimization process and the presence of multiple variables.

Preferably, HNPs with ordered morphologies should be prepared from the co-precipitation method (a wet chemical synthesis), which is simple, efficient, and tractable, in which the size, shape, and even morphology of the particles can be tailored [6]. To obtain well-crystallized HNPs with ordered morphologies, this method strongly depends on the use of additives such as copolymer surfactants (pluronic, F88, F127, P123, and polyvinylpyrrolidone), surface active structures from chelating compounds (thiols-based molecules), and stabilizing agents (triethylamine and (NH_2_)_2_CO of urea), as well as co-precipitating salts (sodium acetate or NaAc) [13,14,15,16]. Unfortunately, the presence of additives significantly affects not only the morphology and percentage yield, but also drastically decreases the HNPs’ purity due to the large amounts of byproducts, such as b-FeOOH, Fe_3_O_4_, or g-Fe_2_O_3_ [17], and the salts’ impurities, such as with ferric and ferrous salts [18]. Alternatively, to overcome such problems, additive-free syntheses of HNPs have been proposed [19], using greener and more benign reagents from biomaterials templates, such as starch. These methods avoid the use of additives, which commonly promote the presence of impurities from the incomplete conversion of the salts’ precursors into HNPs. The use of starch as a template has been reported in a number of previous studies [19]. This includes the use of starch as a template by using starch-coordinated Fe as the mixture of capping agents and, subsequently, the removal of the template by controlled heat treatment. In spite of that, the approach only produces highly agglomerated and disordered hematite macroparticles (greater than 1000 nm) with limited functionality [20]. This is due to the presence of organic remnants, originating from the mixtures of the capping agent used, which is strongly bound to the HNPs. It is well-known that the use of starch-assisted synthesis to obtain well-crystallized HNPs with ordered nanostructured morphologies remains a great challenge.

As mentioned previously, ordered nanostructured HNP morphologies are prepared in various shapes. Notably, HNPs with spherical shapes are regarded as the optimized morphology for high packing density, balanced electron distribution, and charge migration due to its ideal surface-to-volume ratio, which promises good catalytic activity. The intrinsic properties of spherical nanostructured particles can also be fine-tuned by changing certain parameters such as diameter, bulk structure, surface chemistry, chemical composition, and crystallinity, which have led to continuous research interests [21]. Nevertheless, the currently available method for the synthesis of HNPs with a spherical morphology using pluronic surfactant shows that the nanostructure is easily disrupted, leading to the presence of mixed phases such as magnetite, wustite, and hematite with a sharp decrease in Barrett–Emmett–Teller (BET) surface area [22,23]. Evidently, it is challenging to retain spherical-shaped HNPs using the conventional surfactant-templated preparation methods. Therefore, the search for an environmentally friendly route, simple approach, and cost-effective method to prepare stable HNPs with spherical nanostructures for large-scale applications, particularly as a catalyst, is urgently required. To the best of our knowledge, the synthesis of spherical nanostructured HNPs using a native biomaterials template of rice starch without any additives in an aqueous solution has not been reported yet. In continuation of our work on the green synthesis of nanomaterials using rice starch [24], here, we report an additive-free method of rice starch-assisted synthesis for the fabrication of HNPs having a spherical morphology and a size of less than 50 nm. Owing to the spherical shape and porous networks that led to a larger surface area, the HNPs synthesized from this method were successfully utilized as a catalyst for the degradation of methylene blue, a dye contaminant.

## 2. Materials and Methods 

### 2.1. Preparation of Spherical Nanostructured Hematite (Sp-HNP)

All the starting materials were analytical grade and used as received (i.e., without further purification). Rice starch (RS) of 99.9% purity (with ~30% of amylose and ~70% of amylopectin content) was purchased from Euramco (M) Ptd Ltd., Johor, Malaysia. Methylene blue (MB), ethanol, and hydrochloric acid (HCl) were purchased from MERCK (M) Sdn Bhd, Bandar Sunway, Selangor, Malaysia, and acetone was purchased from HmbG Chemical, Ludolfstr., Hamburg, Germany. Iron(II) sulphate heptahydrate (FeSO_4_·7H_2_O, 99.9%) was purchased from Sigma-Aldrich (M) Sdn Bhd, Subang Jaya, Selangor, Malaysia. In a typical RS-assisted synthesis, the HNPs were synthesized according to a calculated molar composition of H_2_O/RS/FeSO_4_·7H_2_O at 1:1:4. Firstly, an appropriate amount of FeSO_4_·7H_2_O and RS were added to double distilled water (<pH = 7), heated to 70 °C, and constantly stirred for 1 h. Then, the mixture was left at room temperature before being filtered, washed with double distilled water, and dried in oven at 100 °C overnight to produce a dark paste. Henceforth, the dark paste is denoted as RS-HNP. Subsequently, the RS-HNP was heated to 700 °C (heating rate of 5 °C/min) in air at 1 atm and maintained at that temperature for 4 h, before slowly being cooled to room temperature. For the complete removal of organic residue, the powder was treated with concentrated HCl (37% *w*/*w*) for 1 h at room temperature before the samples were finally dried and collected as a reddish-brown powder, which is referred to as Sp-HNP. 

### 2.2. Characterization

A Fourier-transform infrared (FTIR) spectrometer (Perkin-Elmer, Spectrum One, Waltham, MA, USA) was used to determine the vibrational wavenumber of the functional groups in the range from 4000 to 450 cm^−1^. The nitrogen adsorption–desorption measurement was performed using an AUTOSORB-1 Quantachrome volumetric adsorption analyzer (Boynton Beach, FL, USA) with nitrogen as the adsorbate at 77.35 K for full-scale adsorption–desorption isotherms. The samples were degassed at 363 K for 3 h and held at 433 K for 12 h before analysis. The specific surface area was calculated using a Barrett–Emmett–Teller (BET) model. Additionally, a Barrett–Joyner–Halenda (BJH) model was used to calculate the pore volume distribution and the average pore size. X-ray diffraction (XRD) was carried out using a Bruker Advance D8 X-ray powder diffractometer (Karlsruhe, Germany) with Cu Kα radiation (λ = 1.54 Å, kV = 40, mA = 40). The morphology of the particles was observed using field emission scanning electron microscopy (FESEM, JSM-6700F, JEOL, Tokyo, Japan), attached with energy dispersive X-ray spectroscopy (FESEM-EDX), operated at 3.0 kV to determine the elemental composition of the samples. The sample was pre-coated with platinum (Pt) at 10^−1^ mbar using a Bio-Rad SEM system. Transmission electron microscopy (TEM) micrographs of the samples were recorded by a JEM-2100F Electron Microscope (JEOL, Tokyo, Japan) at 160 kV accelerated voltage. The samples were ultrasonically dispersed in acetone and trapped on holey carbon membranes. The thermogravimetric analysis (TGA) of the RS-HNP sample was carried out using a Mettler Toledo TGA-SDTA 851e thermal analyzer (Columbus, OH, USA), with a heating rate of 10 °C min^−1^ from room temperature to 1200 °C under a nitrogen atmosphere. For solid samples, the UV-Vis diffuse reflectance spectra (DR UV-Vis) were measured by a Perkin Elmer Lambda 900 ultraviolet-visible/near-infrared (UV-Vis/NIR, Waltham, MA, USA) using a polytetrafluoroethylene polymer as a standard background, scanning in the wavelength ranges from 250 to 900 nm against absorbance. For liquid samples, the UV-Vis spectra were measured using a Thermo Scientific GENESYS 10S (Madison, WI, USA) from 500 to 800 nm.

### 2.3. Catalytic Activity 

Methylene blue (MB), with the chemical formula of C_16_H_18_ClN_3_S, is a thiazine dye commonly used in the textile and printing industries [25]. Based on the structural analysis in Figure S1, the MB molecules consist of thiazines with a heterocyclic center attached to 2 aromatics molecules that provide extra stability and hinder its degradation under visible light. Its industrial effluents are harmful to living organisms and are a major source of water contamination, which is caused by the blocking of sunlight that, in turn, decreases the dissolved oxygen capacity. In humans, MB leads to vomiting, nausea, and permanent damages to eyes on acute exposure [26]. Therefore, the treatment of water effluents containing MB is one of the dire concerns in the field of environmental chemistry. In this study, MB was selected as a model of organic water contaminants to confirm the catalytic degradation activity of the synthesized Sp-HNP. In these experiments, 2.0 mL of 0.1 mM MB was mixed with 1.0 mL of H_2_O_2_ (35% *v*/*v*) in a quartz cuvette and stirred for 30 min in dark conditions to reach the adsorption–desorption equilibrium. Afterwards, 0.05 g of the Sp-HNP was added to the reaction mixture and constantly stirred. The sample was then irradiated with a UV hand lamp (6 W, λ = 365 nm, intensity = 0.8 μW cm^−2^), set perpendicular at a distance of 2 cm. The absorption spectra were recorded through time-dependent UV-Vis at 30-min intervals over the scanning wavelength from 200 to 600 nm, at room temperature (25 ± 2 °C). The absorption band of MB was taken at 506 nm, and the degradation percentage was calculated using the following equation:

Degradation (%) = [(C_o_ − C*_t_*)/(C_o_)] × 100% (1)

where C_o_ is the initial concentration and C*_t_* is the concentration at time *t*.

## 3. Results and Discussion

### 3.1. Characterization of Spherical Hematite (Sp-HNP)

**Figure 1:**
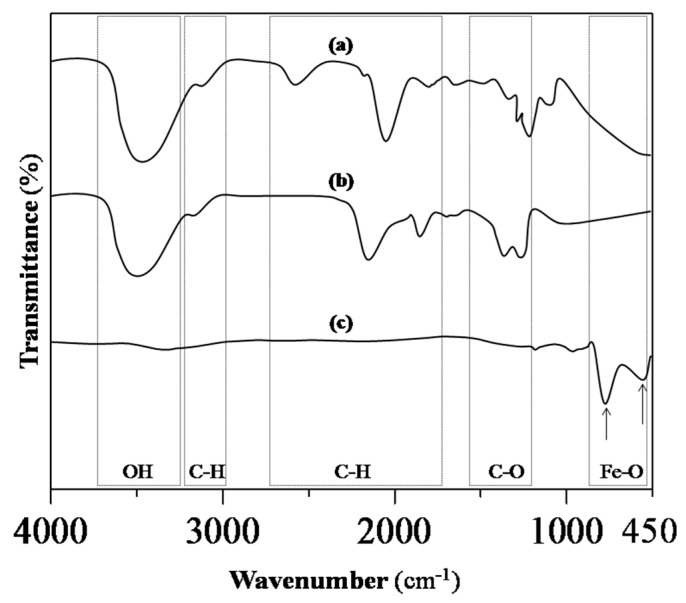
Fourier-transform infrared (FTIR) spectra of the (**a**) rice starch (RS); (**b**) RS-HNP, and (**c**) spherical nanostructured hematite (Sp-HNP).

To monitor the progress of the reaction, FTIR spectroscopy was used to identify the vibrational spectra corresponding to the functional groups of the RS powder, RS-HNP paste, and Sp-HNP, as shown in Figure 1. In Figure 1a, the vibrational band at 2927 cm^−1^ was assigned to C–H stretches, whereas the IR peaks for RS at 1079 and 1021 cm^−1^ were assigned to the anhydroglucose ring of the O–C stretch. Moreover, there was a broad band due to the hydrogen-bonded hydroxyl groups (O–H) at around 3400 cm^−1^, attributed to vibrational stretches of free, intermolecular-, and intramolecular-bound hydroxyl groups [27]. The vibrational bands at 1154, 1079, 1021 and 930 cm^−1^, were attributed to C–O stretching in the fingerprint region [28,29]. Other vibrational bands in the fingerprint region, at 526, 575, 609, 700 and 764 cm^−1^, were due to the skeletal mode vibrations of the pyranose ring in the glucose unit [27,28,29]. Intrinsically, based on the FTIR spectra in Figure 1a, the RS showed vibrational bands of an intact glucose polymer. In the case of the RS-HNP, the FTIR spectra were measured in Figure 1b. It can be observed that the salt precursor regulated by the RS polymer with the presence of Fe-ions’ adsorption from the peak around 1032 cm^−1^, was due to the C–OH bending and vibration of C–O stretching [10,20,28]. In addition, the COOH asymmetric and symmetric stretching bands, which were found around 1623 and 1406 cm^−1^, also supported the presence of Fe-ions–starch [10]. It is suggested that the adsorption of the hematite on the RS involved the interactions with the O–H group attributed to the stretching band at 3420 cm^−1^. The adsorption characteristic of the –FeOOH intermediates was not found at around 905 cm^−1^ due to its low concentration in the mixture of the RS-HNP. It can, therefore, be inferred from Figure 1b that the Fe-ions were absorbed onto the RS polymer through physical interactions and chemical adsorptions from the existence of van der Waals forces [30]. Figure 1c shows the FTIR spectra for the Sp-HNP with strong vibrational bands at 594 and 474 cm^−1^, which were attributed to the hematite, α-Fe_2_O_3_ [10,20]. Judging from the intensity of these two peaks, it can be asserted that band 594 was greater than 474 cm^−1^ at a 3:1 ratio, hence confirming the formation of α-Fe_2_O_3_ [20]. Both the vibrational bands corresponded to the Fe–O bond in the hematite. To clarify, the presence of very weak vibrational bands at 1640 and 3430 cm^−1^ signified the presence of water molecules adsorbed on α-Fe_2_O_3_ due to the hygroscopic nature of the metal oxide [31].

**Figure 2:**
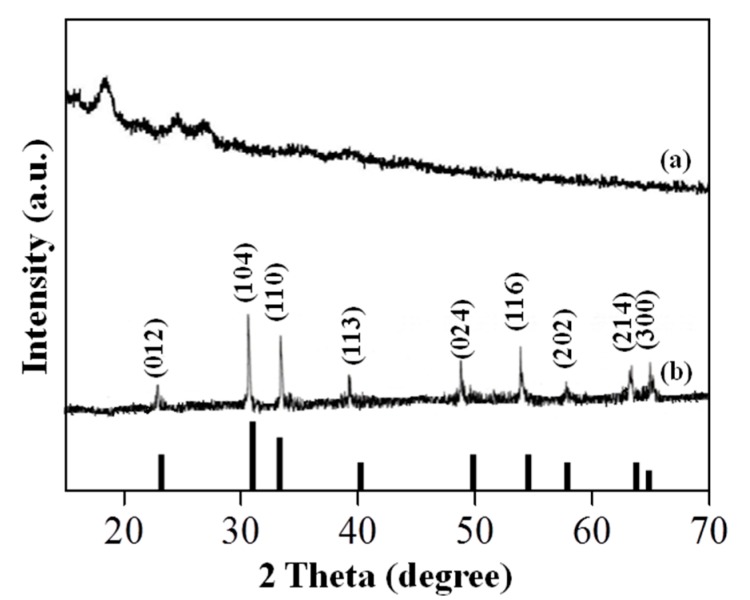
X-ray diffraction (XRD) patterns of the (**a**) RS and (**b**) Sp-HNP, indexed to the rhombohedral structure of hematite JCPDS card No. 33-0664 (vertical lines).

In view of the crystallography, the RS and Sp-HNP were compared using XRD measurements, as shown in Figure 2. It is worth noting that starch is a semi-crystalline biopolymer, containing both amorphous and crystalline structures, with three different crystal types (A, B and C) according to their amylopectin packing [32]. The XRD diffractogram in Figure 2a showed observable peaks for the RS at 16°, 17°, 23° and 24°. The broad and hardly distinguishable diffraction peaks suggested the presence of very small crystallite sizes. Based on the XRD pattern, the RS in its native granules exhibited a typical pattern of the type-A crystal structure [33]. In contrast, the XRD diffractogram for the Sp-HNP showed strong, narrow, sharp, and intense peaks, suggesting that the structures were well crystallized, as shown in Figure 2b. The major XRD peaks were measured at 24.14°, 33.24°, 35.61°, 40.81°, 49.47°, 54.09°, 57.62°, 62.46° and 64.13° and assigned to the (0 1 2), (1 0 4), (1 1 0), (1 1 3), (0 2 4), (1 1 6), (0 1 8), (2 1 4), and (3 0 0) planes, respectively. The diffractogram was indexed to the theoretical data of the pure rhombohedral phase (a hexagonal crystal family) of the hematite (JCPDS card no.33-0664) [10,34] and the lattice parameters: a = b = 0.501 nm, c = 1.373 nm. The powder pattern corresponded to α-Fe_2_O_3_ with all the reflections of the similar peak width. The space group and spinel structure of the Sp-HNP was a R-3c, crystals system which was in good agreement with the literature [35]. Previously, the identical XRD pattern of the obtained α-Fe_2_O_3_ was also reported by others [10,36]. From the XRD analysis, the highly crystalline Sp-HNP suggested that the particles were free from other impurity peaks such as b-FeOOH, Fe_3_O_4_, or g-Fe_2_O_3_ [37]. The high purity indicated that the as-synthesized Sp-HNP is good for catalytic applications. Judging from the intense XRD peaks, it was evident that the Sp-HNP was pure, and the diffraction lines were considerably broadened due to the very small size of the hematite crystals. 

Furthermore, the average particle sizes of the Sp-HNP can be calculated using the Debye–Scherrer equation. Briefly, it gives a relationship between the peak broadening in the XRD and the particle size according to the following equation:

*d* = kλ/(β × cosθ) (2)

where *d* is the particle size of the crystal, k is the Scherrer constant for the shape factor (0.9 was used for common crystallites), λ is the X-ray wavelength used (Cu Kα = 0.15406 nm), β is the full width at the half-height maxima (FWHM) of the concerned XRD peaks, and θ is the Bragg diffraction angle based on the determined incident grazing angle. The three most prominent peaks with Miller indices of (1 0 4), (1 1 0), and (1 1 6) were considered for the estimation of the average crystallite size against Si (1 1 1) FWHM for the reflection (2θ = 28.44° with CuKα radiation) of 2θ = 0.10° as standard. The measured 2θ values for the peaks of the Si (1 1 1) standard materials pattern was in good agreement with the literature values [38]. Using the Debye–Scherrer, Equation (2), the average crystallite sizes of the Sp-HNP were calculated to be in the range of 26–40 nm. 

**Figure 3:**
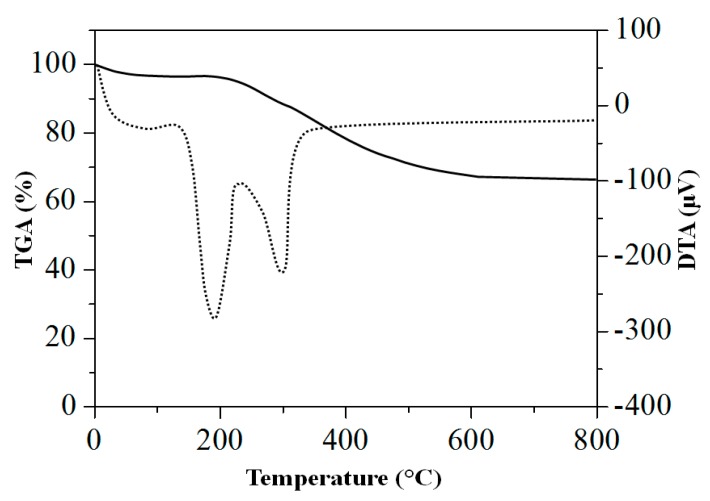
Thermogravimetric-differential thermal analysis (TGA-DTA) thermogram of the RS-HNP.

The representative thermogravimetric-differential thermal analysis (TGA-DTA) thermogram of the RS-HNP is shown in Figure 3. To confirm if the RS-HNP had completely converted into the Sp-HNP, the differential thermal analysis (DTA) thermogram revealed heat variations associated with different endothermic reactions. For the RS-HNP, the mass loss occurred in three steps following the endothermic reactions. Based on the thermogram, the initial weight loss of 2.8% at 110 °C was due to the removal of the adsorbed moisture and other volatile solvents in the RS-HNP. In the second step, a weight loss of 13.8% at 200 °C was measured, indicating the decomposition of an organic component of the polymeric RS in the RS-HNP. The final weight loss of 18.1% after 250 °C corresponded to the removal of the adsorbed oxygen species trapped in the nanoparticles [39]. The final residual mass was found to be 64.7%, which corroborated the presence of α-Fe_2_O_3_ for the Sp-HNP. 

**Figure 4:**
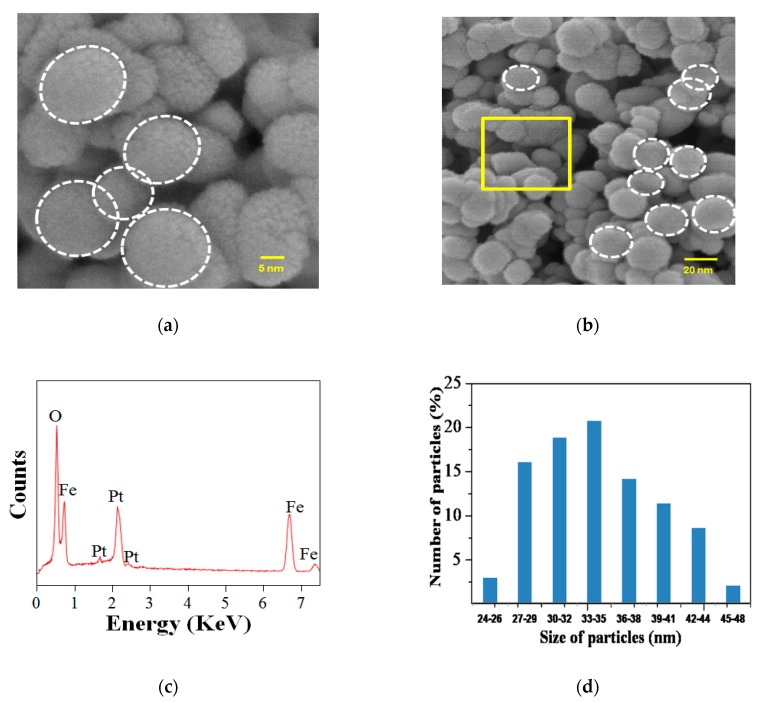
(**a**) Field emission scanning electron microscopy (FESEM) micrograph of the Sp-HNP at 20,000× magnification (the dotted circle suggesting a spherical structure); (**b**) FESEM micrograph of the Sp-HNP at 5000× magnification (with corresponding selected-area electron diffraction (SAED)); (**c**) energy dispersive X-ray spectroscopy (EDX) analysis spectra on SAED; and (**d**) histograms showing the particle size distributions.

Figure 4a,b show the FESEM micrograph for the Sp-HNP, having a spherical morphology, at different magnifications. As suggested in the micrograph images, the Sp-HNP revealed almost monodispersed spherical-shaped nanoparticles. Based on the morphological analysis, the spherical shape of the Sp-HNP was also supported by ImageJ software (version 1.52e, ImageJ2, University of Wisconsin-Madison, WI, USA), as shown in Figure S2. Precisely, the hematite particles were found to nucleate and grow in a uniform direction. The steady growth of the hematite was consistent, which eventually developed well-ordered crystallites nanoparticles and the ordered spherical structure of the Sp-HNP. However, a small agglomeration that affected the Sp-HNP dispersity was deemed present. The composition of the particles was confirmed by the energy dispersive X-ray spectrum (EDX) as shown in Figure 4c. The EDX spectrum of the Sp-HNP indicated that there were only Fe (at 6.5 and 0.7 keV) and O (at 0.2 keV) measured in the samples due to the surface plasmon resonance [40]. This indicated that highly purified Fe_2_O_3_ nanoparticles were prepared by using the RS as a green template. It is worth mentioning that the peaks around 6.5 keV were measured for the binding energies of Fe^3+^ related to the as-synthesized RS-Fe_2_O_3_ colloids [13,41]. As mentioned in the characterization, the Pt element measured at 2.2 keV in the EDX was due to the pre-coating of the sample. As shown in Figure 4d, the Sp-HNP was distributed as spherical nanoparticles with diameters ranging in sizes of 24–48 nm and appeared to be well substantiated with the estimated value of the XRD. Table 1 represents the EDX depth analysis data obtained from selected area in Figure 4b. In Table 1, the elemental composition for the Sp-HNP was confirmed to be Fe (at 35.89%) and O (at 58.27%), with no other impurities being detected. The calculated atomic ratio of Fe/O was about 1:1.62 (Fe_2_O_3.25_), which agreed well with the expected stoichiometry (Fe_2_O_3_). It should be noted that the EDX depth analysis measured several microns in depth from the surface of many HNPs, which might contribute to the experimental uncertainty.

**Figure 5:**
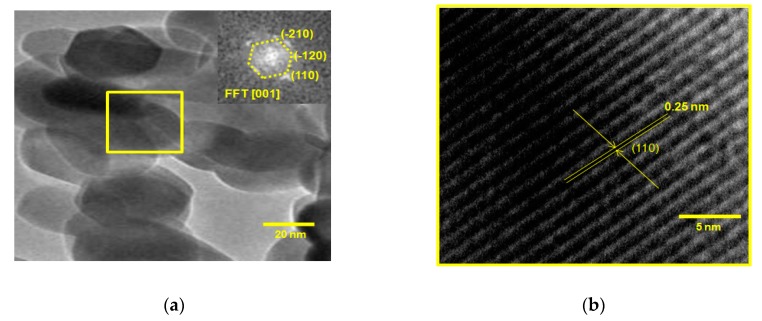
TEM micrograph of (**a**) hematite nanoparticles (HNPs), inset: corresponding fast Fourier-transform (FFT) pattern from the SAED and (**b**) lattice fringes at the (1 1 0) plane.

The TEM micrograph images for the Sp-HNP in Figure 5 confirmed that the spherical nanostructures interlinked over a rhombohedral material [42] to give ordered shape and different sized particles of less than 50 nm. These results strongly supported both the FESEM and XRD measurements. Figure 5a reveals that the nanostructured Sp-HNP was composed of many tiny nanoparticles, with sizes around 20 nm, which self-assembled into regular spheres, which might lead to the formation of a cavity or pores. The regular lattice fringes can be clearly seen in Figure 5b. Based on Figure 5b, the measured interplanar distance or lattice spacing in the TEM image was 0.25 nm, which corresponds to the (1 1 0) plane of the hexagonal phase of Fe_2_O_3_, matching well with the XRD pattern for d_110_ spacing (2θ = 35.61°) of the pure hexagonal hematite. The fast Fourier-transform (FFT) pattern (the inset) of the selected-area electron diffraction (SAED) in Figure 5a indicated the crystalline nature of the Sp-HNP. From the results, it is also suggested that the nanoparticles grew as uniform monocrystals of α-Fe_2_O_3_ without any coalescence effects on the nanoparticles after the removal of the RS by the hydrothermal treatment to obtain the ordered spherical nanostructure. 

**Figure 6:**
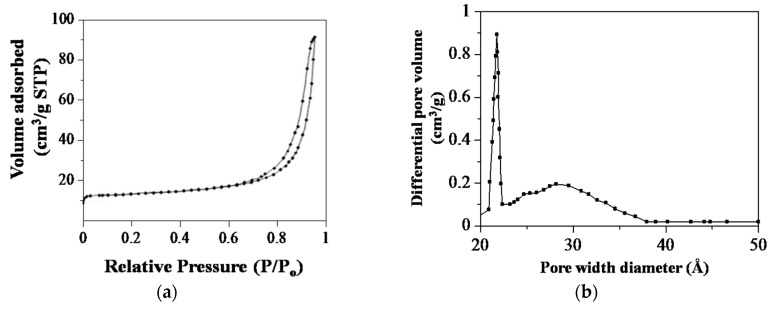
Nitrogen adsorption–desorption measurements (**a**) Isotherms plot; and (**b**) Barrett–Joyner–Halenda (BJH) models.

To quantify the surface area, the nitrogen (N_2_) adsorption–desorption measurements and corresponding pore size distribution (PSD) obtained by the Barrett–Joyner–Halenda (BJH) method for the Sp-HNP are presented in Figure 6. Based on the measurements, the calculated Brunauer–Emmett–Teller (BET) surface area was found to be 20.04 m^2^/g. The obtained surface area for the Sp-HNP was relatively 20 times higher than most natural iron powders (0.1–0.4 m^2^/g) and 4 times higher than the commercial hematite (5 m^2^/g) [43]. Compared with other synthetic hematites, the surface area acquired from the starch-assisted synthesis was also considerably higher than that in the work presented by Park et al. (15.9 m^2^/g) [23] and Wu et al. (15.8 m^2^/g) [44]. According to the International Union of Pure and Applied Chemistry (IUPAC) classification, the N_2_ adsorption–desorption isotherms, as shown in Figure 6a, exhibited a typical type-IV isotherm with a H3-type hysteresis loop (P/P_0_ > 0.6) [45]. The type-IV isotherm was caused by the capillary condensation in the mesopores networks of the Sp-HNP, while the H3-type hysteresis loop was attributed to slit-shaped pores. The results were consistent with those of other works producing HNPs from biomaterials templates [46,47]. As can be seen, the H3-type hysteresis loop appeared in the high-pressure region at a relative pressure higher than 0.6. This proposed the existence of voids, pores, or spaces in extra networks between the interconnected α-Fe_2_O_3_ nanoparticles on the Sp-HNP [48]. It is worth mentioning that the controlled heat treatments and subsequent removal of the RS polymers enabled the synthesis of spatially separated α-Fe_2_O_3_ nanoparticles. As shown in Figure 6b, the Sp-HNP showed PSD at 21–38 Å with predominant pore diameters at 22 Å. Notably, a broad peak centered at 29 Å was attributed to secondary mesopores [44], which were contributed by the Sp-HNP agglomeration.

**Figure 7:**
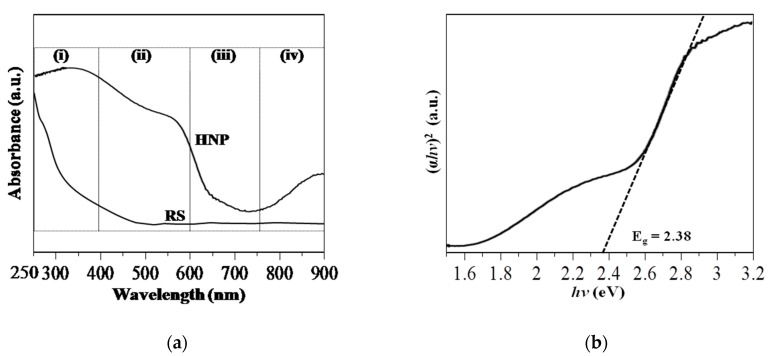
Optical analysis for (**a**) UV-Vis diffuse reflectance spectroscopy (DR UV-Vis) and (**b**) Tauc plot of the Sp-HNP.

The UV-Vis diffuse reflectance spectra of the RS and Sp-HNP samples are shown in Figure 7a. As can be seen, no significant absorption was obtained for the RS sample. Typically, the Sp-HNP gave four absorption regions spectra from (i) to (iv) detected in the UV-Visible spectral region (near-UV and near-infrared) contributed from the α-Fe_2_O_3_ nanoparticles. In the same manner reported previously [43,49], the absorption at 250–400 nm corresponding to the ligand-to-metal charge transfer transitions (LMCT) was measured at region (i), attributed to the Fe–O (π-3d) bond for somewhat from the Fe^3+^ ligand field transitions ^6^A_1_→^4^T_1_(^4^P) at 290–310 nm, ^6^A_1_→^4^E(^4^D) and ^6^A_1_→^4^T_2_(^4^D) at 360–380 nm. For region (ii), the absorption from 400 to 600 nm was attributed to the pair excitation processes ^6^A_1_ + ^6^A_1_→^4^T_1_(^4^G) + ^4^T_1_(^4^G) at 485–550 nm, partially overlapped ligand field transitions at 430 nm on of ^6^A_1_→^4^E, ^4^A_1_(^4^G), and charge-transfer (CT) shoulder. The absorption at 600–750 nm for region (iii) was assigned to the ^6^A_1_→^4^T_2_(^4^G) transition at about 640 nm. Lastly, region (iv) measured from 750 to 900 nm was the ^6^A_1_→^4^T_1_(^4^G) transition at about 900 nm. Both regions (iii) and (iv) were measured for an absorption peak around 500–900 nm, which could be deduced from the Fe–Fe bond on d-to-d transitions [50]. Based on selection rules [49,50], the absorption from the charge-transfer transitions in regions (i) and (ii) was stronger than that from the ligand field transitions in regions (iii) and (iv), as indicated from the UV-Vis absorption intensity [43,49,50]. More importantly, the bandgap (E_g_) value can be estimated using a Tauc Mott plot, by plotting (αhυ)^2^ versus hυ, as shown in Figure 7b. To obtain the desired bandgap, the Tauc’s region was extrapolated to (αhυ)^2^ = 0. The relationship between the absorption coefficient (αhυ)*^n^* and incident photon hυ is shown based on the Tauc formula as follows:

αhυ = A(hυ − E_g_)*^n^* (3)

where α is the absorption coefficient, hυ is the photon energy, A is constant, n can be 2 (indirect transition) or 1/2 (direct transition), and E_g_ is the allowed bandgap. As shown in Figure 7b, the indirect bandgap values were extrapolated at 2.38 eV. The obtained value was comparable to those synthesized α-Fe_2_O_3_ nanoparticles that absorb visible light [51], suggesting that the Sp-HNP has a medium bandgap (at 1–3 eV) and promising semiconductor applications (i.e., as a photocatalyst) [52].

### 3.2. Plausible Formation of Spherical Hematite (Sp-HNP)

**Figure 8:**
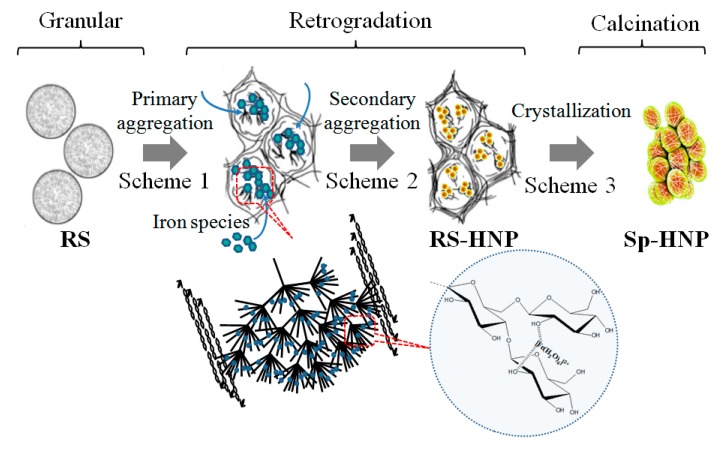
Schematic formation of the Sp-HNP through RS-assisted synthesis.

Based on the characterization of the Sp-HNP, a schematic formation could be depicted in Figure 8. The highlighted additive-free procedure was mainly due to the multifunctional RS that can act as both reducing and capping agents (stabilizer) simultaneously. During the synthesis, the iron(II) sulfate powder initially converted into Fe-ions of metal aquo complex [Fe(H_2_O)_6_]^2+^ upon dissolving in water. Notably, RS consists of linear amylose and branched amylopectin structures, is rich with hydroxyl (OH) backbones, and contains abundant alcohol terminals (COH), as shown in Figure S3. The OH backbones reasonably facilitated the complexation of Fe-ions to the RS polymer, while the alcohol terminals assisted in the reduction of the Fe-ions (+2) to Fe (0) nanoparticles [53]. Taking starch retrogradation process [54] into consideration, the retrograded RS became soluble in hot water, indicating that the polysaccharides granules swelled and burst, destroying the rigidity of the semi-crystalline structure as the smaller amylose started leaching out and leaving behind the amylopectin in the granules. It can, therefore, be inferred that after adding the iron(II) sulfate solution to the RS solution, the Fe-ions were attracted by the oxygen of the OH branches, selectively on the polysaccharides backbone at 70 °C, seeding inside the granules regions of high concentrations of amylopectin. The smaller amylose molecules began to form a gelatinized network on continuous heating outside the granules, which increased the mixture’s viscosity (Scheme 1 in Figure 8). At the same time, the long-chain amylopectin assisted in the nucleation and promoted the initial growth of the hematite crystallites inside the granules, as well as controlling the self-assembly into nano-spherical morphology. For the most part, the van der Waals interactions between the surface molecules of the hematite crystallites (RS-metal ion interactions) are recognized as the driving force for self-assembly, favoring the formation of larger Sp-HNPs spheres due to extensive self-assembled nanocrystals. The formation of the RS-HNP paste indicates the synthesis of starch-stabilized nanoparticles that is similar to other reports for ZnO, Ag, and Au nanoparticles [55,56,57]. In the case of the RS-HNP, the long alkyl chain of RS resulted in stable homogeneous nanoparticles and prevented agglomeration (Scheme 2 in Figure 8). When further heated, the RS-HNP yielded the crystallized Sp-HNP (Scheme 3 in Figure 8) and byproducts of sulfur dioxide, SO_2_, and sulfur trioxide, SO_3_, according to the following chemical equation:

2FeSO_4_ → Fe_2_O_3_ + SO_2_ + SO_3_. (4)

As for the role of RS, it did not only function in reducing and stabilizing the Sp-HNP to protect it from the sintering effect and agglomeration, but it also can be suggested as the nanostructured template to control the morphology, as well as the nanoparticles’ sizes. Intriguingly, the obtained Sp-HNP showed a high-quality hematite, giving a clean spectrum in FTIR, intense XRD diffractogram peaks, and highly ordered nanoparticles in TEM, respectively (Figure 2, Figure 3 and Figure 5). In addition, the biomaterial features of RS have several advantages to its use in a green-synthesis method. First, the environmentally friendly approach from the dispersion of Fe-ions–RS in H_2_O to give the RS-HNP implies benign reagents from biomaterials without the need of organic solvents. Second, the interaction of the van der Waals forces between Fe-ions–RS is relatively weak as compared with the interaction by the hydrogen bond from the typical thiol-based additives [58]. The weak interaction enabled complete removal of the organic component from the metals to produce highly ordered HNPs, as supported by FTIR, TGA, XRD, FESEM-EDX, and TEM analysis. It is of interest that the Sp-HNP showed a magnetic response when applied with an external magnet, as shown in Figure S4. This promises a prospect for easy separation, hence increasing its reusability purpose. Previous work has shown that HNPs have been separated using an external magnet from their reacting mixtures and reused to demonstrate their functional stability [59]. The specific α-Fe_2_O_3_, having rhombohedral repeating units with a hexagonal centered structure and close-packed oxygen in the nano-sized array, allowed the magnetic response [60].

### 3.3. Catalytic Activity

**Figure 9:**
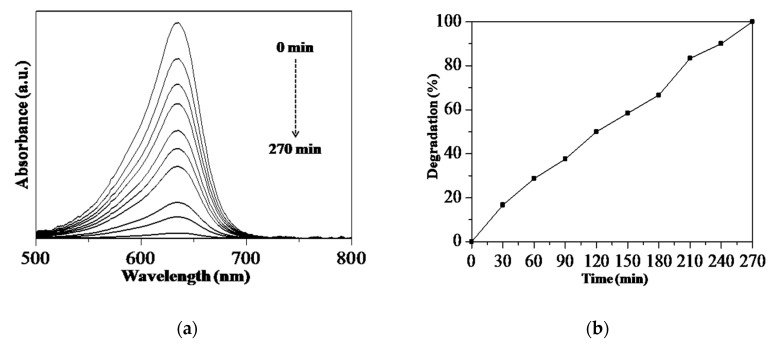
Degradation of methylene blue (MB) for 270 min under (**a**) UV-Vis and (**b**) plot of degradation (%) against time.

Although the focus of the study is the RS assisted-synthesis of the Sp-HNP for nanostructured hematites, the application of the Sp-HNP as a photocatalyst represents a further remarkable feature. The Sp-HNP was expected to demonstrate promising catalytic capability, especially when the spherical morphology can facilitate the optimum charge migration without any diffusion limitations of reactant molecules due to porous networks. As reported by Singh and his co-workers, IONPs were used in the removal of toxic ions and organic pollutants due to the presence of porous networks [61]. In this study, the Sp-HNP underwent a catalytic performance evaluation by comparing the absorption spectra of the degraded MB, which was irradiated under UV light within certain periods. Figure 9 shows the performance of the prepared catalysts, examined by the degradation of MB for 270 min. From Figure 9b, MB was measured to degrade at 17% upon adding H_2_O_2_ exposed under UV light irradiation after 30 min. After a period of time, MB further degraded to 50% and 99%, monitored after 120 and 270 min, respectively. The degradation process was also supported by the photo-captured images in Figure S5a. It is worth mentioning that MB was not significantly degraded in dark conditions, indicating dependency on the UV light. Importantly, the process was chemical photo-degradation rather than physical adsorption. For comparison, the MB degradation experiment was also performed in presence of H_2_O_2_ and/or the UV light without the Sp-HNP (Figure S5). It can be seen that the degradation of MB is hardly initiated without the Sp-HNP, which confirms its catalytic activity. Nonetheless, the catalytic activity of the Sp-HNP is comparable to that of the previously reported HNPs as a catalyst [62]. 

**Figure 10:**
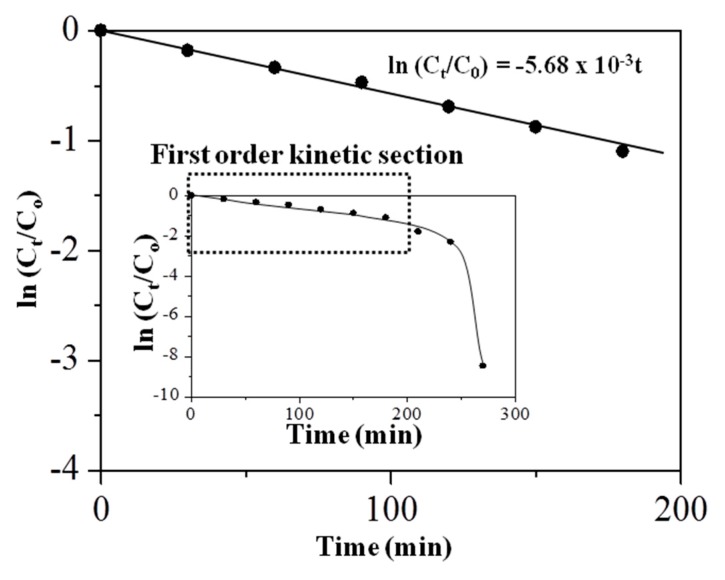
Concentration of MB as a function of the irradiation time for 180 min. The inset shows the concentration of MB for the full irradiation time from 0 to 270 min with the first-order kinetic section defined in the dotted box.

As shown in Figure 10, the rate of the photocatalytic degradation of MB was deduced from the plot of the concentration against the irradiation time for 180 min. The overall MB degradation showed both an exponential and linear relationship of pseudo-first-order kinetics, as indicated in the inset of Figure 10 for the full degradation time up to 270 min. For all the initial concentrations of MB, the degradation gave a straight line that fits well into a logarithmic function, as commonly observed in the photocatalytic degradation reactions of most organic compounds [63]. Hence, the kinetic study on the rate of the degradation for MB in the presence of the Sp-HNP followed the pseudo-first-order reaction. The plot for line (C*_t_*/C_0_) against time showed a linear relationship until 180 min of degradation time, so the rate constant (k) can be determined from the gradient. Here, C_0_ and C*_t_* are the initial concentration of MB and the concentration of MB after *t*, irradiation time, respectively. Moreover, the calculated k for the degradation reactions was 5.68 × 10^−3^ min^−1^ and the half-life, the t_1/2_ value, was found to be 122 min, suggesting that the Sp-HNP causes good photo-degradation of MB.

## 4. Conclusions

In conclusion, ordered nanoparticles and the monodispersed spherical iron oxide of Sp-HNP have been prepared by using an additive-free method, followed by controlled hydrothermal treatments. For the additive-free method, RS was successfully employed as a simple, environmentally friendly and low-cost multifunctional reducing and stabilizing agent. Briefly, the presence of RS protected the nucleation growth of the HNPs against sintering and agglomeration and allowed the avoidance of the use of unnecessary additives. The homogeneous nucleation process of Fe-ions happened during the retrogradation of the rice starch inside the granular networks to regulate the size and morphology of the Sp-HNP. Based on the physicochemical characterization, the synthesized Sp-HNP had a spherical-shaped, nano-sized morphology from 24 to 48 nm, a pure rhombohedral crystalline structure, and a good surface area of 20.04 m^2^/g, which is comparable to most conventional hematite nanoparticles. In addition, because the facile synthesis procedure does not require the introduction of any additives to the reaction system, this simple synthesis method has the potential to be suitable for the synthesis of other novel nanostructured metals. Based on its catalytic performance, the Sp-HNP successfully degraded MB at 99% (after 270 min irradiation under UV light) and obeyed the pseudo-first-order kinetics. In the future, the RS-assisted synthesis presented here will ensure a marked improvement in the studies of advanced nanomaterials that demand spherical nanostructured hematite, especially with respect to size-dependent particles for magnetization properties in biomedical imaging, lithium batteries, and gas sensors. 

## Figures and Tables

**Table 1:** EDX analysis for the spherical hematite (Sp-HNP) with Pt coating.

Elements	Atomic Percentage
Platinum (Pt)	5.47
Oxygen (O)	58.27
Iron (Fe)	35.89

## Supplementary Materials

The following are available online at http://www.mdpi.com/2079-4991/8/9/702/s1, Figure S1: Structural analysis (a) methylene blue (MB) with the chemical formula of C16H18ClN3S; and (b) ball and stick model based on Molecular Mechanics-2 (MM2) for MB at different perspectives showing a sterically hindered central portion of molecules. The presence of Cl- is omitted to avoid bulky structures. Figure S2: Morphological analysis for Sp-HNP showing the spherical morphology on a surface plot analyzed using ImageJ from Java-based software version 1.52e. Figure S3: Rice starch (RS) granules model representing linear amylose and branched amylopectin chains. Figure S4: Photo-captured image on Sp-HNP (reddish-brown powder), showing a magnetic response when applied with an external magnet. Figure S5: Catalytic activity for the degradation of MB (a) Photo-captured images for MB/Sp-HNP/H2O2/UV; Ultraviolet–visible (UV-Vis) spectra for different conditions: (b) MB/UV/H2O2; (c) MB/H2O2; and (d) MB/UV without the presence of the Sp-HNP catalyst
