# Supplementary material for: Additive-Free Rice Starch-Assisted Synthesis of Spherical Nanostructured Hematite for Degradation of Dye Contaminant
Source: Nanomaterials (Basel). 2018 Sep 8;8(9):702. doi: 10.3390/nano8090702 (PMC6163276; doi:10.3390/nano8090702)
Supplement: Supplementary file 1 [file nanomaterials-08-00702-s001.pdf]

Supplementary Materials

Additive-Free Rice Starch-Assisted Synthesis of Spherical Nanostructured Hematite for Degradation of Dye Contaminant

Juan Matmin[1,*], Irwan Affendi[1], Salizatul Ilyana Ibrahim[1], and Salasiah Endud[2]

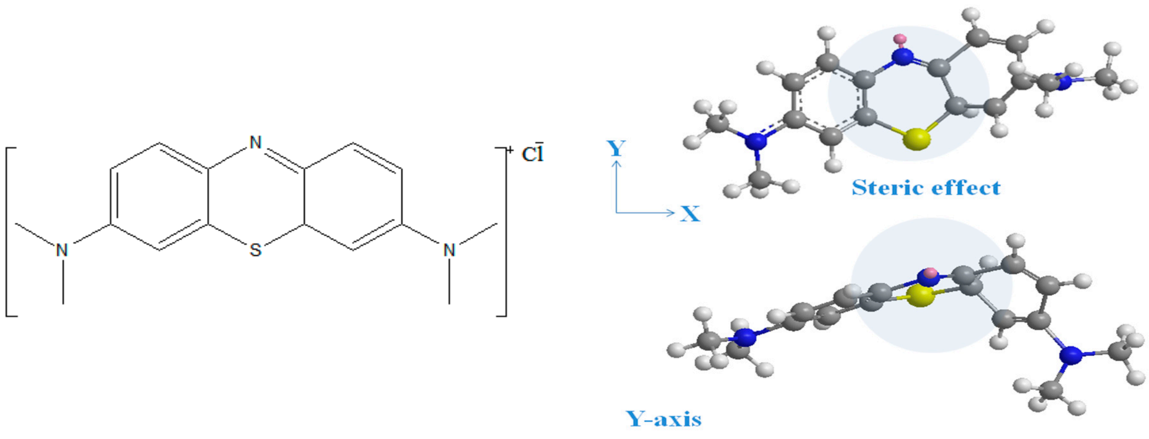

(**a**) (**b**)

Figure S1. Structural analysis (**a**) methylene blue (MB) with the chemical formula of $C_{16}H_{18}ClN_3S$; and (**b**) ball and stick model based on Molecular Mechanics-2 (MM2) for MB at different perspectives showing a sterically hindered central portion of molecules. The presence of Cl^- is omitted to avoid bulky structures.

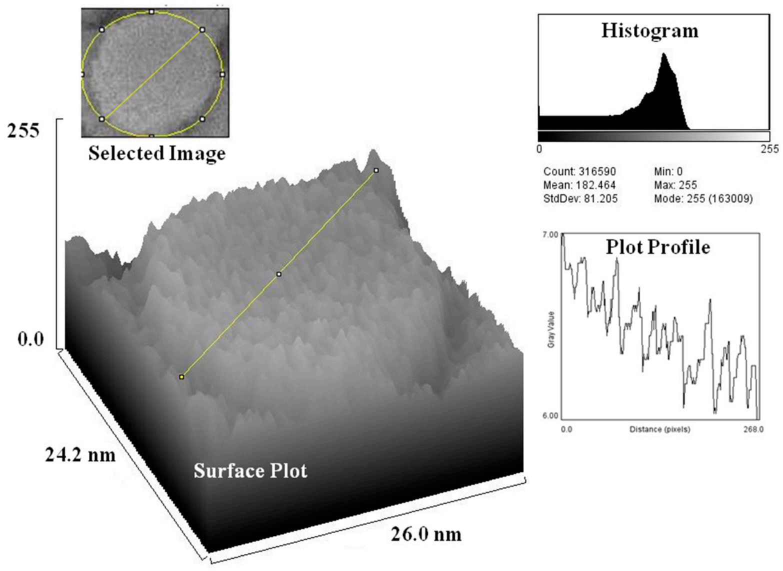

Figure S2. Morphological analysis for Sp-HNP showing the spherical morphology on a surface plot analyzed using ImageJ from Java-based software version 1.52e.

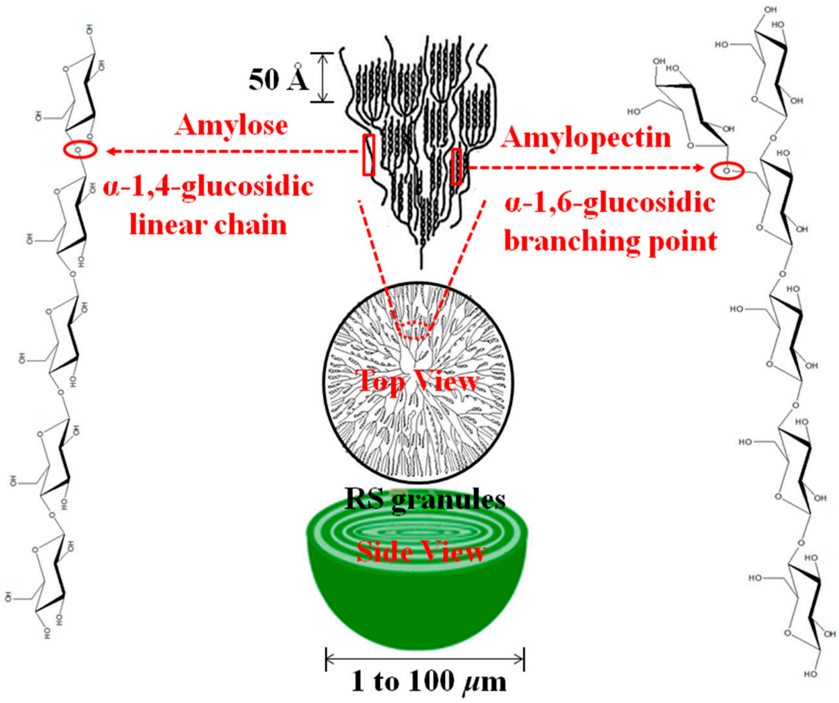

Figure S3. Rice starch (RS) granules model representing linear amylose and branched amylopectin chains.

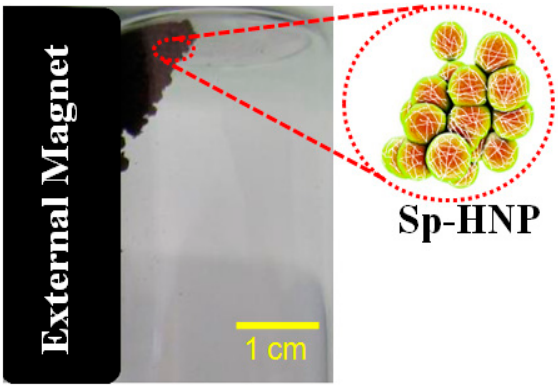

Figure S4. Photo-captured image on Sp-HNP (reddish-brown powder), showing a magnetic response when applied with an external magnet.

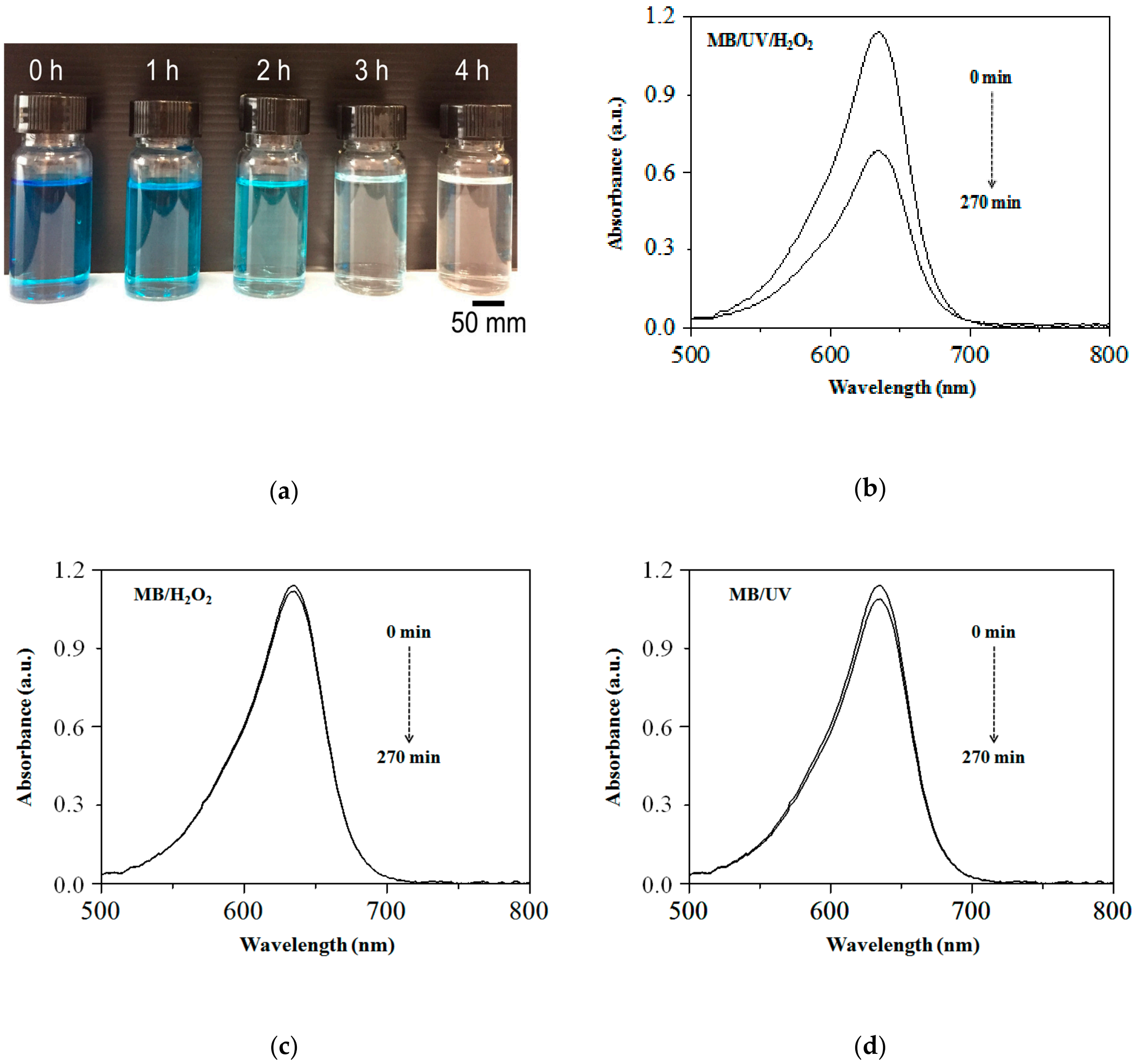

Figure S5. Catalytic activity for the degradation of MB (**a**) Photo-captured images for MB/Sp-HNP/H_2O_2/UV; Ultraviolet–visible (UV–Vis) spectra for different conditions: (**b**) MB/UV/H_2O_2; (**c**) MB/H_2O_2; and (**d**) MB/UV without the presence of the Sp-HNP catalyst
